# Current Perceptions and Improvement Approaches of Pharmaceutical Care Capacity of Community Pharmacists: A Quantitative Analysis Based on Survey Data at Chinese Chain Pharmacies

**DOI:** 10.3390/ijerph17207482

**Published:** 2020-10-14

**Authors:** Zhijia Tang, Pema Lhamu, Hua Ye, Lan Hong, Xiaoqiang Xiang

**Affiliations:** Department of Clinical Pharmacy and Pharmaceutical Affairs, School of Pharmacy, Fudan University, Shanghai 201203, China; zjtang@fudan.edu.cn (Z.T.); 16301030101@fudan.edu.cn (P.L.); yehua@fudan.edu.cn (H.Y.); honglan@fudan.edu.cn (L.H.)

**Keywords:** China, community pharmacist, chain pharmacy, pharmaceutical care, capacity enhancement

## Abstract

*Background:* The benefits of patient-focused pharmaceutical care in improving clinical outcomes and reducing health expenditure have been well documented. However, these services are not fully implemented in many developing countries like China, especially in the community setting at chain pharmacies. *Methods:* A cross-sectional questionnaire-based survey was conducted at nine chain pharmacies in eight provinces of China in late 2019 to assess the difference in attitude, perceived barriers, and expected facilitators of Chinese community pharmacists to deliver pharmaceutical care, as well as their willingness to develop the competencies based on age, education level, and seniority. A quantitative analysis of responses was undertaken by using nonparametric (Kruskal–Wallis) and chi-square tests (followed by Bonferroni post-hoc test). *Results:* A total of 1260 community pharmacists were enrolled in the survey. In total, 16.3% of participant pharmacists often felt that lack of ability had a negative impact on work especially when providing comprehensive medication guidance (39.0%); 44.4% were unsatisfied with academic degrees; 85.8% were “very willing” to improve ability of pharmaceutical care while only 46.9% and 38.4% regarded traditional practice and continuing education as of lots of help, respectively. Main barriers in capacity enhancement were linked to conflict with daily work (35.4%), conflict with family life (21.9%), and poor memories (15.3%). The most welcomed facilitators to enhance pharmaceutical care development included professional skills training (28.4%), self-study of online resources (20.3%), and adult education in universities (15.6%). Community pharmacists of different age, education level, and seniority held significantly different opinions on the above aspects (*p* < 0.05). **Conclusion:** Chinese community pharmacists showed a positive attitude towards capacity enhancement in pharmaceutical care. Specific efforts and reforms should be made to improve the professionalism and to remove barriers based on their age, different education level, and seniority.

## 1. Introduction

Pharmaceutical care has moved the pharmacy profession from primarily focusing on the drug itself to the patient-focused drug therapy and how it should be individualized. The interpretation of pharmaceutical care varies in different countries with diverse culture, professional practice, healthcare delivery, and reimbursement systems [1]. According to the statement of Hepler and Strand in 1990, the term pharmaceutical care was defined as “responsible provision of drug therapy for the purpose of achieving definite outcomes that improve a patient’s quality of life” [2]. Some recent definitions have specified pharmaceutical care as a service provided by pharmacists. The American Society of Hospital Pharmacists (ASHP) regarded pharmaceutical care as one of pharmacists’ missions which contributes to curing diseases, eliminating symptoms, slowing the rate of disease progression, and preventing diseases [3]. In 1998, the International Pharmaceutical Federation (FIP) adopted a more comprehensive definition of pharmaceutical care with emphasis on professional pharmacist–patient relationships, medication records, and collaborative treatment planning with a patient and other healthcare professionals in diverse practice settings. The FIP also claimed that the statement of pharmaceutical care was more useful for pharmacists in developing countries [4]. 

The development of pharmaceutical care in recent decades has shown that it could reduce the risk of potential adverse drug events (ADEs), improve health outcomes and health-related quality of life (HRQOL), and decrease healthcare expenditure [5,6,7]. The community pharmacy is a good place to provide such services as most primary care physicians and hospital pharmacists do not have enough time to provide all of the services that patients require. Community pharmacists are usually more accessible to their patients and, therefore, are in an ideal position to provide regular patient-centered healthcare such as adverse drug reaction monitoring and drug utilization reviews (DUR). Pharmacists have the expertise in detecting, resolving, and preventing medication errors and drug-related problems. Pharmacists’ cognitive services like patient counseling and medication guidance are equally important as the preparation, dispensing, and supply of the drugs themselves [8,9]. Patients would benefit from better adherence to their treatment plan as community pharmacists could identify nonadherence and intervene through education and counseling when supplying medications. Community pharmacists are also well-suited for health screenings to prevent disease and progression and help diagnose new diseases (i.e., type 2 diabetes). Community pharmacy-based point-of-care testing (POCT) such as blood glucose testing and rapid strep testing has shown to help manage chronic disease and acute illness with a good cost-to-benefit ratio, allowing for considerable cost savings for both public and government-funded medical systems [10].

Each country has its own perspective of pharmaceutical care that matches the local situation needs and impacts the implementation and practice model of pharmaceutical care [1]. Overall, the importance has long been recognized in Western countries. A survey conducted by the European Directorate for the Quality of Medicines and HealthCare (EDQM) during 2008 and 2009 showed that nearly 100% of the respondent healthcare professionals agreed that pharmaceutical care would bring good outcomes in terms of patients’ medicine therapy [11]. As to the person who should provide the services, all pharmacists and 89% of other professionals thought the role of pharmacists was either important or specific in delivering pharmaceutical care. In comparison, this practice is still in the dormant stage in China especially in community pharmacy setting. So far, there are 528,610 licensed pharmacists in China, corresponding to 3.8 pharmacists per 10,000 population, among which 90.4% work in community pharmacies. However, the lack of reimbursement mechanism and social status compared to hospital pharmacists has reduced community pharmacists’ willingness to offer pharmaceutical care [12]. A recent study enrolling 163 Chinese community pharmacists showed that they only held moderate understanding of pharmaceutical care and most of them stick to their traditional roles of dispensing prescriptions and selling drugs [13]. 

The limited implementation of pharmaceutical care is a concerning problem all around the world. The 2009 EDQM survey found that only 25% of healthcare professionals regarded the concept of pharmaceutical care as a common practice; only 43% of the patients agreed that such concept was widely used in their country [11]. In China, we suppose there is a similar situation considering the underdeveloped pharmacy education system and pharmacist training system. Another concern is the relatively low requirement of education (a vocational degree) and training time (usually 3 years) to become a community pharmacist in China. According to the data from the Chinese Pharmacists Association, as of July 2018, only 26.7% of licensed pharmacists hold a bachelor’s or higher degree. Therefore, even if with a positive attitude, it is very difficult for Chinese community pharmacists to implement pharmaceutical care due to missing prior training or experience and lack of opportunity to learn. 

This article aims to investigate Chinese community pharmacists’ attitude towards pharmaceutical care, the perceived barriers to providing care, and willingness to improve pharmaceutical care in the future. It was expected that the findings would give Chinese healthcare authorities advice on the support of improving community pharmacist professionalism and removal of the barriers in pharmaceutical care provision.

## 2. Methods

The attitude towards pharmaceutical care, the perceived barriers and facilitators, and level of willingness to improve pharmaceutical care ability of Chinese community pharmacists was assessed through a cross-sectional questionnaire-based survey. Data were collected from nine chain pharmacies in eight provinces/municipalities of China (Beijing, Shanghai, Chongqing, Hubei, Yunnan, Gansu, Xinjiang, and Heilongjiang), with ethical approval (No. SECCR2020-75-01) being achieved as required by local regulation.

### 2.1. Questionnaire/Instrument Design

The questionnaire was written in simplified Chinese. Questions were sorted randomly and modified after the pilot test. The English version and the original simplified Chinese version of the final 12-item, five-section questionnaire are listed in the Supplementary Figure S1. Section A (Question 1, Q2, Q3) collected data on participant pharmacists’ demographics including age, education level, and seniority as a community pharmacist. Section B (Q6, Q7) evaluated the impact of lack of pharmaceutical care skills on work. Section C (Q4, Q8) estimated pharmacists’ subjective motivation. Q4 discussed the willingness to improve pharmaceutical care ability. Q8 emphasized on pharmacists’ satisfaction with their current academic qualifications and future plans to seek a higher degree. Section D (Q5, Q11, and Q12) focused on pharmacists’ previous attempts to obtain pharmaceutical care skills as well as their opinions on the benefits of traditional practice and continuing education. Section E (Q9, Q10) assessed the biggest barrier and expected facilitators of pharmacists to improve pharmaceutical care ability.

### 2.2. Data Collection

The survey was conducted during August–December 2019. All participating chain pharmacies were on the Top 30 Chain Pharmacies with Comprehensive Strength in China list in 2018. Stratified sampling was used at first to sort out chain pharmacies based on a wide geographical distribution across the country, and then volunteer sampling was used to recruit the participants. The sample size was determined by the number of community pharmacists in the region and the model of business (i.e., direct-sale stores or franchisers). All participants were notified of the study purpose and provided informed consent before completing the questionnaire. The questionnaires were distributed both in paper and electronic form in order to boost the response rate. Participants from the same pharmacy received either a paper survey or a web link to submit their answers. The study was also coordinated by the China Association of Pharmaceutical Commerce (CAPC) to have face-to-face interviews with staff pharmacists and managers in order to determine the appropriate way to distribute surveys.

### 2.3. Data Analysis

All questionnaires were screened by two researchers independently. Incomplete (missing >20% questions), obviously wrong (i.e., selected more than one answer in any single-choice questions), and those finished by non-pharmacists were excluded. 

Descriptive statistics were used to summarize the demographic data (Q1–3) and the answers of the multiple-choice question (Q5). The chi-square test was performed to determine if there are statistically significant differences between different groups of age, education level, and seniority (independent variables) on dependent variables. For counts below 5, Fisher’s exact test was used instead. Specifically, for ordinal dependent variables (Q4, Q6, Q11, and Q12), the distribution of answers was compared across the categories using the Kruskal–Wallis test. The gamma coefficient was used to assess the correlation. After the null hypothesis had been rejected, multiple pairwise comparisons were conducted as post-hoc test to determine which pairs of dependent variables are different from one another using a Bonferroni adjustment of the significance level. All data were analyzed with IBM SPSS Statistics for Windows, Version 20.0 (IBM Corp., Armonk, NY, USA). Statistical significance was set as *p* < 0.05.

## 3. Results

### 3.1. Demographics

A total of 1489 questionnaires were recovered, of which 1260 were validated and included in the study. Participants were stratified based on age, education level, and seniority (Table 1). Most participants were 31–45 years old (68.2%), had an associate or lower degree (62.5%), and had been working as a community pharmacist for less than 8 years (83.3%). 

### 3.2. Impact of Incapacity

According to the study, 83.6% of participants often or occasionally felt that they were not capable enough to complete the job well (Table 1). There were statistically significant differences between the young vs. older group (*p* = 0.013), middle-aged vs. older group (*p* = 0.009), low seniority vs. high seniority group (*p* = 0.000), and middle seniority vs. high seniority group (*p* = 0.012) as determined by the Kruskal–Wallis test (Table 2). The median was same among all groups (“occasionally”). The gamma coefficients were less than 0.1 which suggested no association between demographic features and the extent of impacts.

As to what the impacts were, 39.0% and 20.7% participants claimed difficulty in providing comprehensive medication guidance and answering patients’ questions, respectively. The answers were significantly different between age, education level, and seniority groups (*p* < 0.05) (Table 2). As shown in Figure S2, a post-hoc test after the chi-square test revealed that pharmacists with a vocational degree were significantly more likely to fail to get promoted, change jobs, or review prescriptions. However, they were significantly more confident in providing medication guidance and answering patients’ questions. Compared with the middle seniority group, pharmacists with low seniority performed significantly better in answering patients’ questions.

### 3.3. Attitude in Capacity Enhancement

Table 1 showed that 85.8% participants were strongly willing to improve their pharmaceutical care ability. The Kruskal–Wallis test with post-hoc test revealed a significant difference in the level of willingness between young vs. older group (*p* = 0.022), middle-aged vs. older group (*p* = 0.000), and low seniority vs. high seniority group (*p* = 0.000). The median was the same across categories (“very willing”). There was also a weak linear relationship between the age and seniority with the level of willingness suggested by the gamma coefficient (Table 3).

In addition, 44.4% participants were not satisfied with their academic degrees, while 73.3% had planned or were already seeking higher degrees. There were statistically significant differences between all subgroups except for young vs. middle-aged group (*p* = 0.984), as determined by the chi-square test (Table 3). The post-hoc test showed that older pharmacists and those with a bachelor’s or higher degree or high seniority were significantly more satisfied with their degree and did not expect educational promotion. In contrast, middle-aged pharmacists and those with associate degrees were the most dissatisfied group who had a significantly higher proportion in the process of improvement (Figure S2).

### 3.4. Previous Attempts and Current Practice

**Figure 1:**
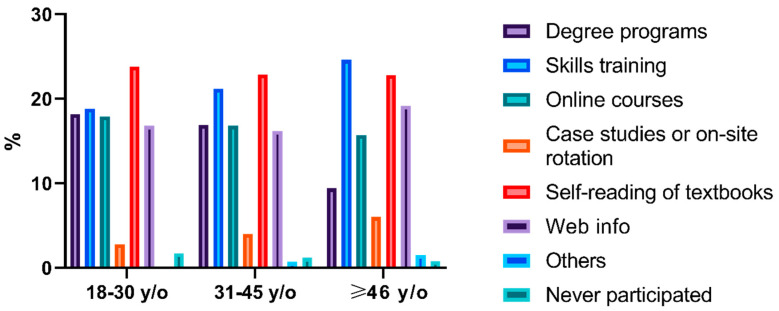
Histogram of capacity enhancement activities based on age.

**Figure 2:**
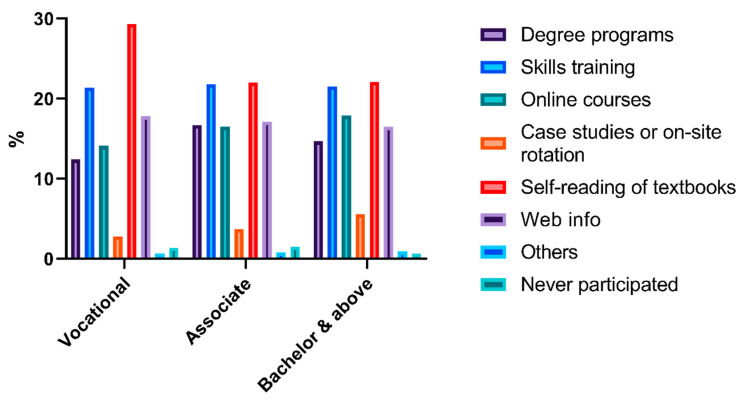
Histogram of capacity enhancement activities based on education level.

**Figure 3:**
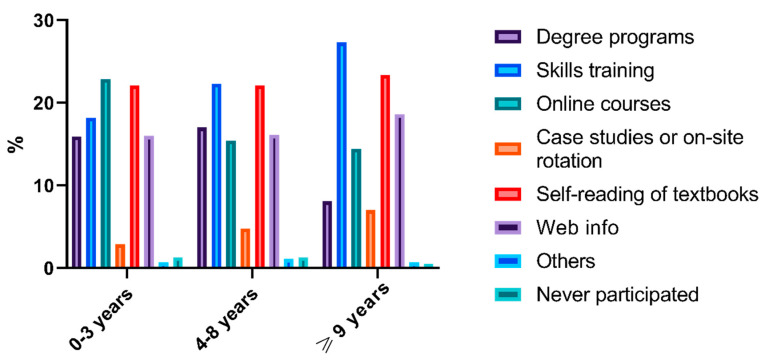
Histogram of capacity enhancement activities based on seniority.

According to the survey, previous attempts to obtain pharmaceutical care skills in the past 3 years included degree programs, skills training, online courses, case studies or on-site rotation, self-reading of textbooks, and web information [Q5]. The histogram of activities shown in Figure 1, Figure 2, Figure 3 indicated that skills training and reading textbooks were the most welcomed activities among all subgroups except the low seniority group who preferred online courses rather than skills training.

Moreover, 3.5% and 5.3% of participants regarded traditional practice (such as dispensing prescriptions and selling drugs) and current continuing education courses offered by the pharmacist association, as of very little or no help, while 49.1% and 55.4% of participants regarded them as of some but limited help (Table 1). For Q11, the Kruskal–Wallis test with post-hoc test showed a significant difference between vocational degree vs. bachelor’s or above degree group (*p* = 0.013), low seniority vs. high seniority group (*p* = 0.014), and middle seniority vs. high seniority group (*p* = 0.009). There was no statistically significant difference between age groups in Q11 (*p* = 0.781). For Q12, the only significant difference occurred between vocational degree vs. bachelor’s or above degree group (*p* = 0.004). The median opinions on the benefits of traditional practice was the same across categories (“some but limited help”), except for pharmacists with a vocational degree in Q11 (“lots of help”). There was no relationship between demographic features and the opinions (Table 4). 

### 3.5. Barriers in Capacity Enhancement

A number of barriers that limited the improvement of pharmaceutical care ability were identified. The main barriers included conflict with daily work (35.4%), conflict with family life (21.9%), and poor memories (15.3%) while 10.2% participants denied any barriers at all (Table 1). According to chi-square test, there were significant differences between all subgroups except low seniority vs. middle seniority group (*p* = 0.429), and middle seniority vs. high seniority group (*p* = 0.225) (Table 5).

As shown by post-hoc test in Figure S2, young pharmacists faced conflicts with daily work and family life significantly more often than their older counterparts. They were also the ones who felt most lost to find ways to improve. The proportion of pharmacists who complained about poor memories was significantly higher in the older and high seniority group. Pharmacists with vocational degree had significantly more conflicts with daily work in ways to improve pharmaceutical care skills. At the same time, pharmacists with a bachelor’s degree or higher had significantly less cost considerations. In contrast, pharmacists of middle age, vocational degree, and seniority between 4 and 8 years had more concerns about the cost of improving.

### 3.6. Facilitators in Capacity Enhancement

The most expected facilitators included professional skills training organized by companies or industry associations (28.4%), self-study of online resources (20.3%), and adult education in universities (15.6%) (Table 1). As shown in Table 6, there were significant differences between all subgroups except low seniority vs. middle seniority group (*p* = 0.090). 

Moreover, according to the post-hoc test in Figure S2, older pharmacists tended to resort to textbooks/publications and online resources significantly more than young pharmacists. Pharmacists with a bachelor’s degree or higher were significantly more willing to participate in public health promotion, while were also the least likely to seek adult education (as in the high seniority group). Compared with other groups, pharmacists with vocational degree were significantly more reluctant to study from online resources or rotate/intern in tertiary hospitals. The low seniority group were the least likely to participate in public health promotion and read textbooks/publications, and significantly less prone to rely on online resources and rotation/internships to develop pharmaceutical care capabilities.

## 4. Discussion

This is the first nationwide and quantitative study to comprehensively investigate the attitude of Chinese community pharmacists towards pharmaceutical care, the obstacles and facilitating factors they recognized, and their attempts and willingness to improve based on age, education level, and seniority. As such, it provided valuable insights into the role, education system, and financial, social, and policy support structures of community pharmacists in China.

As mentioned above, 83.6% of community pharmacists admitted that lack of pharmaceutical care ability had negatively affected their work. However, we observed an anomaly, that is, less-educated pharmacists did worse in reviewing prescriptions, but they were significantly more confident in providing medication guidance and answering patients’ questions compared to higher degree groups. This anomaly may be explained by the changes in Chinese pharmacy education in recent decades. Since the number of vocational schools offering pharmacy degrees has largely declined especially in urban areas of China, those pharmacists with low education levels may be the same group of pharmacists working in rural and less populated communities, and therefore are more familiar with their regular patients. No similar situation was observed between different age or seniority groups. 

Generally, Chinese community pharmacists had a very positive attitude towards improving pharmaceutical care ability and nearly half of the participants expressed dissatisfaction with their degrees. Pharmacists of middle age (31–45 years old) and middle education level (associate) were the most dissatisfied with the degree and had the largest proportion of being in the process of academic progress. This coincides with the time point when the first bachelor’s degree program in clinical pharmacy was approved by the Ministry of Education of China in 2006 [14]. Thereafter, the number of colleges and universities offering clinical pharmacy education increased (51 in 2020). However, with a total of approximately 2000 bachelor graduates each year, it is far from adequate to meet the country’s needs. In addition, the lower wages and social status of community pharmacies compared to hospitals or pharmaceutical companies further exacerbated the outflow of talents to other industries. As a result, most community pharmacists have relatively low degrees (associate or vocational degree) and graduated from related majors such as nursing, nutrition, and biology. Lack of professional training in clinical pharmacy prevents them from understanding the true meaning of pharmaceutical care and how to provide the services. It is worth mentioning that the minimum education requirements to sit for a pharmacist licensing exam in China has been moved to the associate degree starting in 2021, and applicant’s major is restricted to pharmacy-related majors, which heralds a future of more professional community pharmacists with higher academic degrees. Further changes to the Chinese pharmacy education system and pharmacist training system are necessary to improve community pharmacists’ professionalism in providing pharmaceutical care. Establishing the Doctor of Pharmacy (Pharm.D.) program and modifying the undergraduate curriculum to make it more focused on pharmaceutical care and pharmacotherapy may be two feasible ways to solve this problem.

Less than half of community pharmacists believed that they were given adequate opportunities and conditions to develop their professionalism in pharmaceutical care. Only 46.9% and 38.4% of participants said traditional practice (i.e., dispensing) and continuing education was of lots of help, respectively. This may be explained by three reasons. Firstly, since almost every hospital has its own outpatient pharmacy, prescription outflows are minimal in China. Very few patients choose to fill prescriptions at community pharmacies, which deprives community pharmacists’ opportunities to practice pharmaceutical care. Secondly, nearly all community pharmacies are run by profit-oriented companies. The remuneration of pharmacists is determined by how many products are sold, so they are encouraged to spend their time selling over-the-counter medicines and health foods, rather than providing free counseling services. New models of remuneration and reward need to be developed to support the provision of pharmaceutical care in the community setting. Thirdly, despite the minimum hours of continuing education required for license renewal, most of the accredited continuing education courses are a repetition of book knowledge, and the learning efficiency is low. Developing more attractive online courses and live seminars can help pharmacists improve their service capabilities outside the workplace.

Transition towards pharmaceutical care services also requires the support from regulatory agencies. The EDQM survey in 2009 implied that lack of a legal basis or a contract with health insurance may hinder the implementation of pharmaceutical care [11]. At present, there is no pharmacist law in China, which means there are no hard rules on whether pharmacists should provide pharmaceutical care. To make things worse, neither the Chinese national medical insurance program nor commercial insurance companies reimburse for pharmacists’ cognitive services. As a result, it is difficult for community pharmacists to proactively provide pharmaceutical care for legal or financial incentives. The regional and national medical authorities and pharmacist associations may set up policy documents or contracts to integrate community pharmacists into the primary care team with other healthcare professionals so that they can work together to improve patient outcomes [15]. On the other hand, the Chinese government has a strong commitment to the regulatory framework for pharmacy practice, which is demonstrated by the release of the first draft Licensed Pharmacists Law by the Chinese National Health Commission in 2017, and the second draft was just posted for comments in June 2020. 

In some countries like the US, pharmaceutical care is inherently part of community pharmacists’ routine work. However, they are more regarded as sellers than healthcare providers in China. Long-term interpersonal relationships between the pharmacist and patient are rare. The public, including the prescribing physicians, does not expect them to deliver high-quality cognitive services. Therefore, the focus should not be only on enacting new laws just because they allow pharmacists to obtain financial benefits when providing services, as public acceptance could be jeopardized. Issues such as the responsibilities of pharmacists under the law, the value of pharmaceutical care in decreasing drug-related problems, and the benefits of patients to cooperate with pharmacists should be emphasized. Better communication channels and awareness should be established to improve the public’s recognition and appreciation of the contributions of pharmaceutical care.

Regarding the barriers for improving pharmaceutical care skills, most participants identified time/financial pressures and work–family conflict. Paid training and holiday/bonus compensation may be effective, especially among pharmacists of middle age, vocational degree, and seniority between 4 and 8 years. 

The most welcomed facilitators included professional skills training, online resources, and adult education. The Chinese Pharmacists Association should take the responsibility to provide high-quality continuing education and training, either on-site or online. Again, paid training and cash (or other) rewards for academic progress may do the trick. Specifically, a mutual agreement on rotation/internships between community pharmacies and tertiary hospitals may be helpful for pharmacists with middle-high education level and seniority. The health promotion program (i.e., smoking cessation) is another option for highly educated pharmacists to engage and empower individuals and communities to achieve optimal health results. 

## 5. Strengths and Limitations

This study provided important information on the attitude, perceived barriers, and expected facilitators of Chinese community pharmacists to deliver pharmaceutical care, and their attempts and willingness to improve pharmaceutical care ability. The advantage of research lies in the quantitative method, enabling the collection of rich data that provided valuable insights and in-depth understandings of the role, education system, and support of Chinese community pharmacists. It is also the first nationwide survey that recruited over 1200 community pharmacists from eight provinces.

One of the limitations of this study is the high proportion of middle-aged pharmacists included, which may bias the results. This limitation was partly addressed by the stratification of respondents according to age, education level, and seniority, as well as the use of post-hoc test. In addition, since the questionnaire was self-reported, when asked about their willingness and satisfaction (Q4, Q11, and Q12), respondents may tend to give a positive answer. Lastly, when evaluating frequency using an ordinal scale (Q6), different respondents may have different definition criteria.

## 6. Conclusions

Overall, Chinese community pharmacists have shown a positive attitude towards improving the ability of pharmaceutical care. Both healthcare authorities at all levels and pharmacist associations should make specific efforts and reforms to improve the professionalism and to remove barriers that vary with age, education level, and seniority.

## Figures and Tables

**Table 1:** Community pharmacists’ answers to the questionnaire (*n* = 1260).

Characteristics	No. of Pharmacists, *n* (%)	Characteristics	No. of Pharmacists, *n* (%)
**Q1. Age**		** Q8. Academic degrees**	
18–30 y/o (young)	138 (11.0%)	Satisfied, no plans	207 (16.4%)
31–45 y/o (middle-aged)	859 (68.2%)	Satisfied, with further plan	435 (34.5%)
≥46 y/o (older)	263 (20.9%)	Unsatisfied, in plan now	312 (24.8%)
**Q2. Education level**		Unsatisfied, have plans but were not implemented	177 (14.0%)
Vocational	183 (14.5%)	Unsatisfied, no plans	71 (5.6%)
Associate	605 (48.0%)	Never thought of	57 (4.5%)
Bachelor or above	471 (37.4%)		
**Q3. Seniority**		**Q9. Biggest barriers**	
0–3 years (low)	644 (51.1%)	Cost	70 (5.6%)
4–8 years (middle)	406 (32.2%)	Conflict with daily work	446 (35.4%)
≥9 years (high)	210 (16.7%)	Conflict with family life	276 (21.9%)
**Q4. Willingness**		Poor memories	193 (15.3%)
Very willing	1081 (85.8%)	Do not know how to improve	83 (6.6%)
A bit willing	156 (12.4%)	No motivation or stimulus	41 (3.3%)
Not very willing	4 (0.3%)	Others	22 (1.7%)
Definitely not willing	1 (0.1%)	No barriers	128 (10.2%)
Never thought of	17 (1.3%)		
**Q5. Previous attempts**		**Q10. Facilitators**	
Degree programs	521 (41.3%)	Adult education	197 (15.6%)
Skills training	733 (58.2%)	Skills training	358 (28.4%)
Online courses	567 (45.0%)	Online resources	256 (20.3%)
Case studies or on-site rotation	146 (11.6%)	Textbooks/publications	132 (10.5%)
Self-reading of textbooks	778 (61.7%)	Case-study	97 (7.7%)
Web info	574 (45.6%)	Rotation/internship	112 (8.9%)
Others	28 (2.2%)	New drug info	42 (3.3%)
Never participated	40 (3.2%)	Public health promotion	40 (3.2%)
		Others	6 (0.5%)
		Never thought of	20 (1.6%)
**Q6. Frequency of feeling incapable**		**Q11. Benefits of traditional practice**	
Very often	205 (16.2%)	Lots of help	590 (46.9%)
Occasionally	849 (67.4%)	Some but limited help	617 (49.1%)
Seldom	168 (13.3%)	Very little/no help	44 (3.5%)
Never	38 (3.0%)	Never thought of	6 (0.5%)
**Q7. Impact of insufficient abilities**		**Q12. Benefits of continuing education**	
Review prescriptions	47 (3.7%)	Lots of help	483 (38.4%)
Medication guidance	491 (39.0%)	Some but limited help	697 (55.4%)
Answer questions	261(20.7%)	Very little/no help	66 (5.3%)
Communicate	167 (13.3%)	Never participated	13 (1.0%)
Job promotion	62 (4.9%)		
Find other jobs	35 (2.8%)		
Others	26 (2.1%)		
None	171 (13.6%)		

**Table 2:** Differences between age, education level, and seniority groups for Q6 (frequency of feeling incapable) and Q7 (impact of insufficient abilities).

	Characteristics	Groups		Test Value	Degrees of Freedom	*p*	Gamma Coefficient
Q6	Age	All		11.298	2	0.004	0.001 (*p* = 0.990)
		18–30 y/o vs.	31–45 y/o	−29.771		0.897	
			≥46 y/o	92.459		0.013	
		31–45 y/o vs.	≥46 y/o	62.687		0.009	
	Education level	All		1.031	2	0.597	−0.015 (*p* = 0.771)
		Vocational vs.	Associate	*		*	
			Bachelor or above	*		*	
		Associate vs.	Bachelor or above	*		*	
	Seniority	All		21.809	2	0.000	−0.032 (*p* = 0.494)
		0–3 years vs.	4–8 years	−37.589		0.148	
			≥9 years	111.134		0.000	
		4–8 years vs.	≥9 years	73.546		0.012	
Q7	Age	All		35.429	14	0.001	
		18–30 y/o vs.	31–45 y/o	8.854	7	0.263	
			≥46 y/o	24.089	7	0.001	
		31–45 y/o vs.	≥46 y/o	21.753	7	0.003	
	Education level	All		60.288	14	0.000	
		Vocational vs.	Associate	15.870	7	0.026	
			Bachelor or above	57.776	7	0.000	
		Associate vs.	Bachelor or above	28.024	7	0.000	
	Seniority	All		27.746	14	0.015	
		0–3 years vs.	4–8 years	12.145	7	0.096	
			≥9 years	16.320	7	0.022	
		4–8 years vs.	≥9 years	13.088	7	0.070	

* No post-hoc test was conducted as the null hypothesis was not rejected (*p* > 0.05).

**Table 3:** Differences between age, education level, and seniority groups for Q4 (willingness to improve) and Q8 (satisfaction of academic degrees).

	Characteristics	Groups		Test Value	Degrees of Freedom	*p*	Gamma Coefficient
Q4	Age	All		16.937	2	0.000	0.210 (*p* = 0.011)
		18–30 y/o vs.	31–45 y/o	0.790		1.000	
			≥46 y/o	59.757		0.022	
		31–45 y/o vs.	≥46 y/o	60.547		0.000	
	Education level	All		0.474	2	0.789	0.028 (*p* = 0.699)
		Vocational vs.	Associate	*		*	
			Bachelor or above	*		*	
		Associate vs.	Bachelor or above	*		*	
	Seniority	All		15.956	2	0.000	0.252 (*p* = 0.001)
		0–3 years vs.	4–8 years	−27.980		0.105	
			≥9 years	64.957		0.000	
		4–8 years vs.	≥9 years	36.977		0.118	
Q8	Age	All		90.519	10	0.000	
		18–30 y/o vs.	31–45 y/o	0.679	5	0.984	
			≥46 y/o	34.696	5	0.000	
		31–45 y/o vs.	≥46 y/o	84.650	5	0.000	
	Education level	All		303.985	10	0.000	
		Vocational vs.	Associate	111.628	5	0.000	
			Bachelor or above	136.084	5	0.000	
		Associate vs.	Bachelor or above	101.449	4	0.000	
	Seniority	All		120.590	10	0.000	
		0–3 years vs.	4–8 years	23.304	5	0.000	
			≥9 years	112.496	5	0.000	
		4–8 years vs.	≥9 years	41.217	5	0.000	

* No post-hoc test was conducted as the null hypothesis was not rejected (*p* > 0.05).

**Table 4:** Differences between age, education level, and seniority groups for Q11 (benefits of traditional practice) and Q12 (benefits of continuing education).

	Characteristics	Groups		Test Value	Degrees of Freedom	*p*	Gamma Coefficient
Q11	Age	All		0.494	2	0.781	−0.037 (*p* = 0.491)
		18–30 y/o vs.	31–45 y/o	*		*	
			≥46 y/o	*		*	
		31–45 y/o vs.	≥46 y/o	*		*	
	Education level	All		8.601	2	0.014	0.135 (*p* = 0.004)
		Vocational vs.	Associate	−44.103		0.293	
			Bachelor or above	−78.757		0.013	
		Associate vs.	Bachelor or above	34.654		0.230	
	Seniority	All		9.863	2	0.007	−0.090 (*p* = 0.054)
		0–3 years vs.	4–8 years	8.049		1.000	
			≥9 years	71.986		0.014	
		4–8 years vs.	≥9 years	80.035		0.009	
Q12	Age	All		0.482	2	0.786	0.037 (*p* = 0.493)
		18–30 y/o vs.	31–45 y/o	*		*	
			≥46 y/o	*		*	
		31–45 y/o vs.	≥46 y/o	*		*	
	Education level	All		11.026	2	0.004	−0.004 (*p* = 0.924)
		Vocational vs.	Associate	−45.839		0.257	
			Bachelor or above	−87.532		0.004	
		Associate vs.	Bachelor or above	41.692		0.096	
	Seniority	All		1.559	2	0.459	0.057 (*p* = 0.223)
		0–3 years vs.	4–8 years	*		*	
			≥9 years	*		*	
		4–8 years vs.	≥9 years	*		*	

* No post-hoc test was conducted as the null hypothesis was not rejected (*p* > 0.05).

**Table 5:** Differences between age, education level, and seniority groups for Q9 (barriers).

	Characteristics	Groups		Test Value	Degrees of Freedom	*p*
Q9	Age	All		190.030	14	0.000
		18–30 y/o vs.	31–45 y/o	26.870	7	0.000
			≥46 y/o	82.572	7	0.000
		31–45 y/o vs.	≥46 y/o	140.828	7	0.000
	Education level	All		33.102	14	0.003
		Vocational vs.	Associate	14.103	7	0.049
			Bachelor or above	22.102	7	0.002
		Associate vs.	Bachelor or above	16.423	7	0.022
	Seniority	All		25.899	14	0.027
		0–3 years vs.	4–8 years	6.997	7	0.429
			≥9 years	23.627	7	0.001
		4–8 years vs.	≥9 years	9.397	7	0.225

**Table 6:** Differences between age, education level, and seniority groups for Q10 (facilitators).

	Characteristics	Groups		Test Value	Degrees of Freedom	*p*
Q10	Age	All		362.678	22	0.000
		18–30 y/o vs.	31–45 y/o	22.416	9	0.008
			≥46 y/o	31.725	9	0.000
		31–45 y/o vs.	≥46 y/o	23.332	9	0.005
	Education level	All		528.675	22	0.000
		Vocational vs.	Associate	22.543	9	0.007
			Bachelor or above	68.818	9	0.000
		Associate vs.	Bachelor or above	46.342	9	0.000
	Seniority	All		501.457	22	0.000
		0–3 years vs.	4–8 years	15.019	9	0.090
			≥9 years	37.919	9	0.000
		4–8 years vs.	≥9 years	18.021	9	0.035

## Supplementary Materials

The following are available online at https://www.mdpi.com/1660-4601/17/20/7482/s1, Figure S1: Questionnaire, Figure S2: Post hoc results.
