# Supplementary material for: Current Perceptions and Improvement Approaches of Pharmaceutical Care Capacity of Community Pharmacists: A Quantitative Analysis Based on Survey Data at Chinese Chain Pharmacies
Source: Int J Environ Res Public Health. 2020 Oct 14;17(20):7482. doi: 10.3390/ijerph17207482 (PMC7602434; doi:10.3390/ijerph17207482)
Supplement: Supplementary file 1 [file ijerph-17-07482-s001.zip › Supplementary 2. Post hoc results.docx]

**Figure S2. The results of Bonferroni post hoc test of Q7, Q8, Q9, and Q10**

| **Q7: Impact * Age Crosstabulation** | | | | | |
| --- | --- | --- | --- | --- | --- |
| % within Age | | | | | |
|  | | Age | | | Total |
|  |  | 18-30 y/o | ≥46 y/o | 31-45 y/o |  |
| Impact | Answer questions | 18.8%_a_ | 20.0%_a_ | 25.1%_a_ | 23.3% |
|  | Communicate | 21.0%_a_ |  |  | 2.6% |
|  | Find other jobs | 2.9%_a_ | 4.2%_a_ | 2.8%_a_ | 3.1% |
|  | Job promotion | 4.3%_a_ | 3.8%_a_ | 6.3%_a_ | 5.5% |
|  | Medication guidance | 39.1%_a_ | 42.9%_a_ | 44.9%_a_ | 43.8% |
|  | None | 8.0%_a_ | 21.7%_b_ | 14.5%_a_ | 15.2% |
|  | Others | 0.7%_a_ | 4.6%_a_ | 1.9%_a_ | 2.3% |
|  | Review prescriptions | 5.1%_a_ | 2.9%_a_ | 4.4%_a_ | 4.2% |
| Total | | 100.0% | 100.0% | 100.0% | 100.0% |
| Each subscript letter denotes a subset of Age categories whose column proportions do not differ significantly from each other at the .05 level. | | | | | |

| **Q7: Impact * Education_level Crosstabulation** | | | | | |
| --- | --- | --- | --- | --- | --- |
| % within Education_level | | | | | |
|  | | Education_level | | | Total |
|  |  | Associate | Bachelor & above | Vocational |  |
| Impact | Answer questions | 21.6%_a_ | 27.6%_a_ | 9.8%_b_ | 20.7% |
|  | Communicate |  |  | 9.2%_b_ | 2.2% |
|  | Find other jobs | 2.9%_a_ | 4.7%_a_ | 11.9%_b_ | 5.7% |
|  | Job promotion | 6.6%_a_ | 1.0%_b_ | 21.0%_c_ | 8.3% |
|  | Medication guidance | 47.6%_a_ | 45.5%_a_ | 21.4%_b_ | 40.5% |
|  | None | 15.5%_a_ | 15.7%_a, b_ | 9.5%_b_ | 14.1% |
|  | Others | 2.1%_a_ | 0.2%_b_ | 1.4%_a, b_ | 1.3% |
|  | Review prescriptions | 3.7%_a_ | 5.2%_a_ | 15.9%_b_ | 7.2% |
| Total | | 100.0% | 100.0% | 100.0% | 100.0% |
| Each subscript letter denotes a subset of Education_level categories whose column proportions do not differ significantly from each other at the .05 level. | | | | | |

| **Q7: Impact * Seniority Crosstabulation** | | | | | |
| --- | --- | --- | --- | --- | --- |
| % within Seniority | | | | | |
|  | | Seniority | | | Total |
|  |  | 0-3 yrs | ≥9 yrs | 4-8 yrs |  |
| Impact | Answer questions | 17.7%_a_ | 25.3%_a, b_ | 28.3%_b_ | 22.0% |
|  | Communicate | 14.1%_a_ |  |  | 7.7% |
|  | Find other jobs | 2.6%_a_ | 6.3%_b_ | 1.7%_a_ | 3.0% |
|  | Job promotion | 5.4%_a_ | 3.2%_a_ | 6.0%_a_ | 5.2% |
|  | Medication guidance | 41.8%_a_ | 40.5%_a_ | 41.4%_a_ | 41.5% |
|  | None | 11.8%_a_ | 18.9%_b_ | 16.9%_a, b_ | 14.4% |
|  | Others | 2.2%_a_ | 2.1%_a_ | 2.3%_a_ | 2.2% |
|  | Review prescriptions | 4.3%_a_ | 3.7%_a_ | 3.4%_a_ | 4.0% |
| Total | | 100.0% | 100.0% | 100.0% | 100.0% |
| Each subscript letter denotes a subset of Seniority categories whose column proportions do not differ significantly from each other at the .05 level. | | | | | |

| **Q8: Satisfaction * Age Crosstabulation** | | | | | |
| --- | --- | --- | --- | --- | --- |
| % within Age | | | | | |
|  | | Age | | | Total |
|  |  | 18-30 y/o | ≥46 y/o | 31-45 y/o |  |
| Satisfaction | Never thought of | 2.9%_a_ | 17.6%_b_ | 4.0%_a_ | 6.7% |
|  | Satisfied, no plan | 13.1%_a_ | 39.0%_b_ | 21.2%_a_ | 23.7% |
|  | Satisfied, with further plan | 34.3%_a_ |  |  | 5.4% |
|  | Unsatisfied, has plan but not implemented | 15.3%_a_ | 17.0%_a_ | 22.6%_a_ | 20.3% |
|  | Unsatisfied, in plan now | 29.9%_a_ | 17.0%_b_ | 43.5%_c_ | 35.8% |
|  | Unsatisfied, no plan | 4.4%_a_ | 9.3%_a_ | 8.7%_a_ | 8.2% |
| Total | | 100.0% | 100.0% | 100.0% | 100.0% |
| Each subscript letter denotes a subset of Age categories whose column proportions do not differ significantly from each other at the .05 level. | | | | | |

| **Q8: Satisfaction * Education_level Crosstabulation** | | | | | |
| --- | --- | --- | --- | --- | --- |
| % within Education_level | | | | | |
|  | | Education_level | | | Total |
|  |  | Associate | Bachelor & above | Vocational |  |
| Satisfaction | Never thought of | 5.5%_a_ | 9.9%_a_ | 4.4%_a_ | 6.6% |
|  | Satisfied, no plan | 15.9%_a_ | 48.3%_b_ | 7.1%_c_ | 23.9% |
|  | Satisfied, with further plan |  |  | 23.5%_b_ | 5.0% |
|  | Unsatisfied, has plan but not implemented | 22.3%_a_ | 16.0%_a_ | 22.4%_a_ | 20.4% |
|  | Unsatisfied, in plan now | 46.6%_a_ | 20.5%_b_ | 33.9%_c_ | 36.0% |
|  | Unsatisfied, no plan | 9.7%_a_ | 5.3%_a_ | 8.7%_a_ | 8.2% |
| Total | | 100.0% | 100.0% | 100.0% | 100.0% |
| Each subscript letter denotes a subset of Education_level categories whose column proportions do not differ significantly from each other at the .05 level. | | | | | |

| **Q8: Satisfaction* Seniority Crosstabulation** | | | | | |
| --- | --- | --- | --- | --- | --- |
| % within Seniority | | | | | |
|  | | Seniority | | | Total |
|  |  | 0-3 yrs | ≥9 yrs | 4-8 yrs |  |
| Satisfaction | Never thought of | 2.2%_a_ | 19.1%_b_ | 6.2%_c_ | 5.5% |
|  | Satisfied, no plan | 10.9%_a_ | 45.4%_b_ | 28.2%_c_ | 19.8% |
|  | Satisfied, with further plan | 34.1%_a_ |  |  | 21.0% |
|  | Unsatisfied, has plan but not implemented | 14.8%_a_ | 15.6%_a, b_ | 23.2%_b_ | 17.0% |
|  | Unsatisfied, in plan now | 32.3%_a_ | 12.8%_b_ | 33.2%_a_ | 29.9% |
|  | Unsatisfied, no plan | 5.8%_a_ | 7.1%_a_ | 9.3%_a_ | 6.8% |
| Total | | 100.0% | 100.0% | 100.0% | 100.0% |
| Each subscript letter denotes a subset of Seniority categories whose column proportions do not differ significantly from each other at the .05 level. | | | | | |

| **Q9: Impact * Age Crosstabulation** | | | | | |
| --- | --- | --- | --- | --- | --- |
| % within Age | | | | | |
|  | | Age | | | Total |
|  |  | 18-30 y/o | ≥46 y/o | 31-45 y/o |  |
| Impact | Conflict with daily work | 38.4%_a_ | 22.0%_b_ | 28.7%_a, b_ | 28.3% |
|  | Conflict with family life | 21.0%_a_ | 9.5%_b_ | 27.4%_a_ | 22.9% |
|  | Cost | 9.4%_a, b_ | 5.7%_b_ | 11.5%_a_ | 10.1% |
|  | Do not know how to improve | 15.2%_a_ | 7.6%_b_ | 8.2%_b_ | 8.8% |
|  | No barriers | 8.0%_a_ | 4.9%_a_ | 6.1%_a_ | 6.0% |
|  | No motivation or stimulus | 5.8%_a_ | 4.5%_a_ | 4.4%_a_ | 4.6% |
|  | Others |  | 1.1%_a_ | 1.0%_a_ | 1.0% |
|  | Poor memories | 2.2%_a_ | 44.7%_b_ | 12.7%_c_ | 18.3% |
| Total | | 100.0% | 100.0% | 100.0% | 100.0% |
| Each subscript letter denotes a subset of Age categories whose column proportions do not differ significantly from each other at the .05 level. | | | | | |

| **Q9: Impact * Education_level Crosstabulation** | | | | | |
| --- | --- | --- | --- | --- | --- |
| % within Education_level | | | | | |
|  | | Education_level | | | Total |
|  |  | Associate | Bachelor & above | Vocational |  |
| Impact | Conflict with daily work | 29.1%_a_ | 31.1%_a_ | 18.6%_b_ | 28.3% |
|  | Conflict with family life | 24.1%_a_ | 21.7%_a_ | 21.9%_a_ | 22.9% |
|  | Cost | 11.6%_a_ | 6.6%_b_ | 14.2%_a_ | 10.1% |
|  | Do not know how to improve | 6.9%_a_ | 10.9%_a_ | 9.8%_a_ | 8.8% |
|  | No barriers | 6.3%_a_ | 6.2%_a_ | 4.9%_a_ | 6.0% |
|  | No motivation or stimulus | 3.6%_a_ | 6.0%_a_ | 4.4%_a_ | 4.6% |
|  | Others | 0.7%_a_ | 1.1%_a_ | 1.6%_a_ | 1.0% |
|  | Poor memories | 17.7%_a_ | 16.6%_a_ | 24.6%_a_ | 18.3% |
| Total | | 100.0% | 100.0% | 100.0% | 100.0% |
| Each subscript letter denotes a subset of Education_level categories whose column proportions do not differ significantly from each other at the .05 level. | | | | | |

| **Q9: Impact * Seniority Crosstabulation** | | | | | |
| --- | --- | --- | --- | --- | --- |
| % within Seniority | | | | | |
|  | | Seniority | | | Total |
|  |  | 0-3 yrs | ≥9 yrs | 4-8 yrs |  |
| Impact | Conflict with daily work | 30.0%_a_ | 23.3%_a_ | 28.3%_a_ | 28.4% |
|  | Conflict with family life | 23.2%_a_ | 23.8%_a_ | 21.9%_a_ | 22.9% |
|  | Cost | 9.8%_a_ | 9.0%_a_ | 11.1%_a_ | 10.1% |
|  | Do not know how to improve | 10.3%_a_ | 4.8%_b_ | 8.6%_a, b_ | 8.8% |
|  | No barriers | 6.5%_a_ | 5.2%_a_ | 5.7%_a_ | 6.0% |
|  | No motivation or stimulus | 5.0%_a_ | 5.2%_a_ | 3.7%_a_ | 4.6% |
|  | Others | 0.8%_a_ | 1.4%_a_ | 1.0%_a_ | 1.0% |
|  | Poor memories | 14.5%_a_ | 27.1%_b_ | 19.7%_a, b_ | 18.3% |
| Total | | 100.0% | 100.0% | 100.0% | 100.0% |
| Each subscript letter denotes a subset of Seniority categories whose column proportions do not differ significantly from each other at the .05 level. | | | | | |

| **Q10: Expected_assistance * Age Crosstabulation** | | | | | |
| --- | --- | --- | --- | --- | --- |
| % within Age | | | | | |
|  | | Age | | | Total |
|  |  | 18-30 y/o | ≥46 y/o | 31-45 y/o |  |
| Expected_assistance | Adult Education | 26.1%_a_ | 17.1%_a_ | 24.6%_a_ | 23.5% |
|  | Case-study | 6.5%_a_ |  |  | 1.1% |
|  | Never thought of |  | 4.4%_b_ | 2.4%_a, b_ | 2.4% |
|  | New drug info | 5.8%_a_ | 7.0%_a_ | 4.2%_a_ | 5.0% |
|  | Online resources | 18.8%_a_ | 31.6%_b_ | 33.1%_b_ | 30.5% |
|  | Others |  | 0.6%_a_ | 0.9%_a_ | 0.7% |
|  | Public health promotion | 3.6%_a_ | 8.2%_a_ | 4.0%_a_ | 4.8% |
|  | Rotation/internship | 11.6%_a_ | 8.9%_a_ | 15.1%_a_ | 13.3% |
|  | Skills training | 17.4%_a_ |  |  | 2.9% |
|  | Textbooks/publications | 10.1%_a_ | 22.2%_b_ | 15.6%_a, b_ | 16.0% |
| Total | | 100.0% | 100.0% | 100.0% | 100.0% |
| Each subscript letter denotes a subset of Age categories whose column proportions do not differ significantly from each other at the .05 level. | | | | | |

| **Q10: Expected_assistance * Education_level Crosstabulation** | | | | | |
| --- | --- | --- | --- | --- | --- |
| % within Education_level | | | | | |
|  | | Education_level | | | Total |
|  |  | Associate | Bachelor & above | Vocational |  |
| Expected_assistance | Adult Education | 27.3%_a_ | 13.0%_b_ | 26.8%_a_ | 22.6% |
|  | Case-study |  |  | 2.7%_b_ | 0.6% |
|  | Never thought of | 2.9%_a_ | 2.2%_a_ | 1.1%_a_ | 2.3% |
|  | New drug info | 4.9%_a_ | 5.4%_a_ | 3.8%_a_ | 4.8% |
|  | Online resources | 30.0%_a_ | 35.4%_a_ | 19.1%_b_ | 29.4% |
|  | Others | 0.7%_a_ | 0.4%_a_ | 1.1%_a_ | 0.7% |
|  | Public health promotion | 3.7%_a_ | 8.3%_b_ | 1.1%_a_ | 4.6% |
|  | Rotation/internship | 15.4%_a_ | 16.2%_a_ | 2.2%_b_ | 12.9% |
|  | Skills training |  |  | 32.8%_b_ | 6.9% |
|  | Textbooks/publications | 15.1%_a, b_ | 19.1%_b_ | 9.3%_a_ | 15.2% |
| Total | | 100.0% | 100.0% | 100.0% | 100.0% |
| Each subscript letter denotes a subset of Education_level categories whose column proportions do not differ significantly from each other at the .05 level. | | | | | |

| **Q10: Expected_assistance * Seniority Crosstabulation** | | | | | |
| --- | --- | --- | --- | --- | --- |
| % within Seniority | | | | | |
|  | | Seniority | | | Total |
|  |  | 0-3 yrs | ≥9 yrs | 4-8 yrs |  |
| Expected_assistance | Adult Education | 19.7%_a_ | 10.2%_b_ | 22.3%_a_ | 19.2% |
|  | Case-study | 6.7%_a_ |  |  | 4.2% |
|  | Never thought of | 0.9%_a_ | 6.2%_b_ | 2.3%_a, b_ | 1.9% |
|  | New drug info | 3.9%_a_ | 6.2%_a_ | 3.5%_a_ | 4.1% |
|  | Online resources | 20.5%_a_ | 29.7%_a, b_ | 33.6%_b_ | 24.9% |
|  | Others | 0.6%_a_ |  | 0.8%_a_ | 0.6% |
|  | Public health promotion | 2.3%_a_ | 8.6%_b_ | 5.5%_b_ | 3.9% |
|  | Rotation/internship | 9.0%_a_ | 18.8%_b_ | 11.7%_a, b_ | 10.9% |
|  | Skills training | 28.0%_a_ |  |  | 17.5% |
|  | Textbooks/publications | 8.4%_a_ | 20.3%_b_ | 20.3%_b_ | 12.8% |
| Total | | 100.0% | 100.0% | 100.0% | 100.0% |
| Each subscript letter denotes a subset of Seniority categories whose column proportions do not differ significantly from each other at the .05 level. | | | | | |
