# Supplementary material for: Current Perceptions and Improvement Approaches of Pharmaceutical Care Capacity of Community Pharmacists: A Quantitative Analysis Based on Survey Data at Chinese Chain Pharmacies
Source: Int J Environ Res Public Health. 2020 Oct 14;17(20):7482. doi: 10.3390/ijerph17207482 (PMC7602434; doi:10.3390/ijerph17207482)
Supplement: Supplementary file 1 [file ijerph-17-07482-s001.zip › Supplementary 1. Questionnaire.docx]

**Appendix 1a. Questionnaire on pharmaceutical care for Chinese community pharmacists**

| **Items** | **Answers** |
| --- | --- |
| Q1: Your age is | 1. 18-30 years old 2. 31-45 years old 3. ≥46 years old |
| Q2: Your current highest degree is | 1. Vocational 2. Associate 3. Bachelor or above |
| Q3: Your seniority as a community pharmacist is | 1. 0-3 years 2. 4-8 years 3. ≥9 years |
| Q4: Do you want to further improve your pharmaceutical care ability? | 1. Very willing 2. A bit willing 3. Not very willing 4. Definitely not willing 5. Never thought of |
| Q5: In the past three years, what activities have you participated in to improve pharmaceutical care skills? (Multiple choices) | 1. Associate or bachelor degree programs 2. Skills training provided by companies, government, or industry associations 3. Online courses 4. Various forms of case studies or on-site rotation 5. Self-reading of textbooks 6. Searching on the internet 7. Others (please specify _____) 8. I have not participated in any enhancement activities. |
| Q6: How often do you feel that you are not capable enough to complete the job well? | 1. Very often 2. Occasionally 3. Seldom 4. Never |
| Q7: What is the biggest problem your lack of pharmaceutical care skills brings to your work? | 1. Cannot review the prescriptions properly 2. Cannot provide comprehensive medication guidance 3. Unable to answer patients’ questions 4. Cannot communicate with patients smoothly or cannot get patients’ trust 5. Lose promotion opportunities 6. Hard to find other jobs 7. Other (please specify ____) 8. Basically no problems |
| Q8: Are you satisfied with your current educational level? Is there any plan to further improve it? | 1. Satisfied, and I have no plans in the near future. 2. Satisfied, but I have further plans. 3. Unsatisfied, and I am in the process of improvement. 4. Unsatisfied, there are corresponding plans but have not been implemented. 5. Unsatisfied, but I have no plans in the near future. 6. Never thought of |
| Q9: What is the biggest barrier for you to improve pharmaceutical care ability? | 1. Cost 2. Conflict with daily work 3. Conflict with family life 4. Poor memories 5. Do not know how to improve 6. No motivation or stimulus 7. Others (please specify _____) 8. No barriers |
| Q10: What is the most expected learning ways for you to improve pharmaceutical care ability? | 1. Adult education in universities 2. Professional skills training organized by companies or industry associations 3. Self-study of online resources 4. Self-study of pharmacy textbooks/publications 5. Case-study with physicians or hospital pharmacists 6. Rotation/internship in third-tier hospitals 7. Obtain information on newly marketed drugs 8. Participate in public health promotion activities 9. Others (please specify _____) 10. Never thought of |
| Q11: Did traditional work (i.e., dispensing prescriptions, selling drugs) help improve your pharmaceutical care skills? | 1. There is a lot of help. 2. There is some help. 3. There is very little or no help. 4. Never thought of |
| Q12: Did continuing education (CE) organized by the Pharmacist Association help improve your pharmaceutical care skills? | 1. There is a lot of help. 2. There is some help. 3. There is very little or no help. 4. I have not participated in any continuing education. |

**Appendix 1b. Questionnaire on pharmaceutical care for Chinese community pharmacists (simplified Chinese)**

| **问题** | **选项** |
| --- | --- |
| 1．您的年龄是 | 1. 30岁及以下 2. 31-45岁 3. 46岁及以上 |
| 2. 您目前最高的学历是 | 1. 中专 2. 高职或大专 3. 本科及以上 |
| 3. 您注册为执业药师至今有 | 1. 3年及以内 2. 4-8年 3. 9年及以上 |
| 4．从您内心来说，是否希望自己的专业能力有进一步的提升 | 1. 非常希望 2. 有点希望 3. 不太希望 4. 不希望 5. 没想过 |
| 5. 近三年来，您曾经参加过的能力提升活动有（可多选） | 1. 参加专科、本科等的学历教育班 2. 参加公司、政府、或行业协会的技能培训 3. 参加网校提供的在线培训课程 4. 参与病例研讨或现场实践 5. 自己阅读专业书籍 6. 自己查询网站上的药学专业知识 7. 其他（请注明________________） 8. 没有参加过 |
| 6．在您的工作经历中，是否因为感到自己能力不足，难以胜任工作 | 1. 经常有 2. 偶尔有 3. 几乎没有 4. 从来没有 |
| 7. 您认为，执业能力不足给您工作带来的最大困扰是 | 1. 无法正确审核调配医师处方 2. 无法为消费者提供全面的用药指导 3. 无法解答消费者提出的专业问题 4. 与消费者难以进行沟通并取得信任 5. 学历不高，导致没有晋升机会 6. 找别的单位或工作岗位很难 7. 其他困扰（请注明_______________） 8. 基本没有困扰 |
| 8．您对目前的学历是否满意？是否有进一步提升学历的计划？ | 1. 满意，短期内没有提升计划 2. 满意，但如果有可能还想继续提升学历 3. 不满意，正在提升学历过程中 4. 不满意，有相应提升计划但未实施 5. 不满意，但短期内没有提升计划 6. 没有考虑过 |
| 9．您在学历/能力提升过程中遇到的最大的困难是 | 1. 学习费用过高，无法承受 2. 与本职工作时间冲突 3. 家中有老人小孩需要照顾 4. 本人年龄偏大记忆力不好 5. 不知道有哪些途径可以提升 6. 没有学习和提升的动力 7. 其他（请注明________________） 8. 没有遇到过困难 |
| 10．您目前最希望得到提升自己执业能力的方式是 | 1. 接受高等学校的成人学历教育 2. 参加公司或行业协会组织的专业技能培训 3. 利用网络的课程教学资源自学 4. 阅读正规出版的各类药学专业书籍 5. 参加医学药学专家主办的病例研讨 6. 在三甲医院药房短期实习 7. 企业的新上市药品相关知识培训 8. 参与公益宣传和科普讲座等的机会 9. 其他（请注明_____________） 10. 没考虑过 |
| 11．实际上，您这些年在日常药品销售中，专业能力也能够得到提升 | 1. 确实可以有很大提升 2. 能够提升，但很有限 3. 没觉得有什么提升 4. 没想过 |
| 12．您认为每年（执业）药师协会组织的执业药师继续教育对自己的能力提升 | 1. 有非常大的帮助 2. 有一些帮助 3. 没什么帮助或完全没帮助 4. 没参加过，或者没有考虑过 |
